# Neuropsychiatric Symptoms in Rhombencephalosynapsis: A Clinical Report

**DOI:** 10.1007/s12311-024-01740-8

**Published:** 2024-09-04

**Authors:** Dennis J.L.G. Schutter, Dan Doherty, James O. Phillips, Avery H. Weiss, Roderick P.P.W.M. Maas

**Affiliations:** 1 Department of Experimental Psychology, Helmholtz Institute, Utrecht University, Heidelberglaan 1, Utrecht, 3584 CS the Netherlands; 2 Department of Pediatrics, University of Washington School of Medicine, Seattle, USA; 3 Department of Otolaryngology, University of Washington School of Medicine, Seattle, USA; 4 Department of Ophthalmology, University of Washington School of Medicine, Seattle, USA; 5 Department of Neurology, Donders Institute for Brain, Cognition, and Behaviour, Radboud University Medical Center, Nijmegen, the Netherlands

**Keywords:** Cerebellar Cognitive Affective Syndrome, Emotion, Executive Functioning, Psychopathology, Vermis

## Abstract

Rhombencephalosynapsis (RES) is a hindbrain malformation characterized by a missing cerebellar vermis with apposition or fusion of the cerebellar hemispheres. The present clinical case report provides a comprehensive, longitudinal overview of cognitive and affective manifestations in a 22-year-old patient with RES. The patient shows clinical signs of emotional reactivity and dysregulation, impulsivity, and impairments in executive functioning since early childhood. These features fit the constellation of neuropsychiatric symptoms observed in patients with congenital and acquired abnormalities of the posterior vermis. It is proposed that patients with RES may show affective and cognitive difficulties which increase their vulnerability to psychological stress and risk of developing mental health issues.

## Introduction

Rhombencephalosynapsis (RES) is a midline brain malformation involving partial or complete agenesis of the cerebellar vermis, fusion of the cerebellar hemispheres, merging of the dentate nuclei, and rarely fusion of the superior cerebellar peduncles [1]. RES can be isolated or occur in association with other congenital anomalies, such as in Gómez-López-Hernández syndrome (GLHS) [2, 3]. GLHS is a rare congenital neurocutaneous syndrome characterized by RES, partial bilateral hair loss (alopecia), distinct craniofacial features (e.g., abnormal head shape and posterior angulated ears), and, in a subgroup of patients, trigeminal anaesthesia [4, 5]. The aetiology of RES is still largely unknown. In addition to a wide range of motor symptoms, which may include developmental delays, ataxia, swallowing difficulties, muscular hypotonia, abnormal eye movements, and “figure of eight” head-nodding stereotypy [6], cognitive and emotional disturbances occur in varying degrees of severity [6, 7]. The collection of cognitive and emotional disturbances associated with cerebellar pathology is known as the cerebellar cognitive affective syndrome (CCAS) or Schmahmann’s syndrome [8–10]. CCAS is characterized by deficits in one or more of the following four domains: (i) executive functions, (ii) visuospatial cognition, (iii) linguistic processing and (iv) affect regulation.

Longitudinal studies on psychological functioning and wellbeing from childhood to early adulthood in RES remain scarce. Here, we describe a 22-year-old white woman with RES and bilateral parietal alopecia who underwent neuropsychological evaluations and brain imaging on several occasions during her life.

### Clinical Information

She was delivered at 39-weeks gestational age by caesarean section following an uncomplicated pregnancy, born to non-consanguineous parents. There was no family history of developmental delay, intellectual disability, autism, hydrocephalus, cerebral palsy, seizures, other birth defects, or neurological conditions in general. Maternal grandmother developed post-traumatic epilepsy in her 30s after a neck injury. Medical records documented the presence of psychiatric symptoms, including anxiety, depression, attention deficits, and hyperactivity on the maternal side of the family.

Prenatal ultrasound in the ninth month of pregnancy revealed a dilated left lateral ventricle. Additional ultrasound scans during the first 6 months postpartum confirmed the unilateral ventriculomegaly. No other significant neurological findings were observed during this period.

**Fig. 1:**
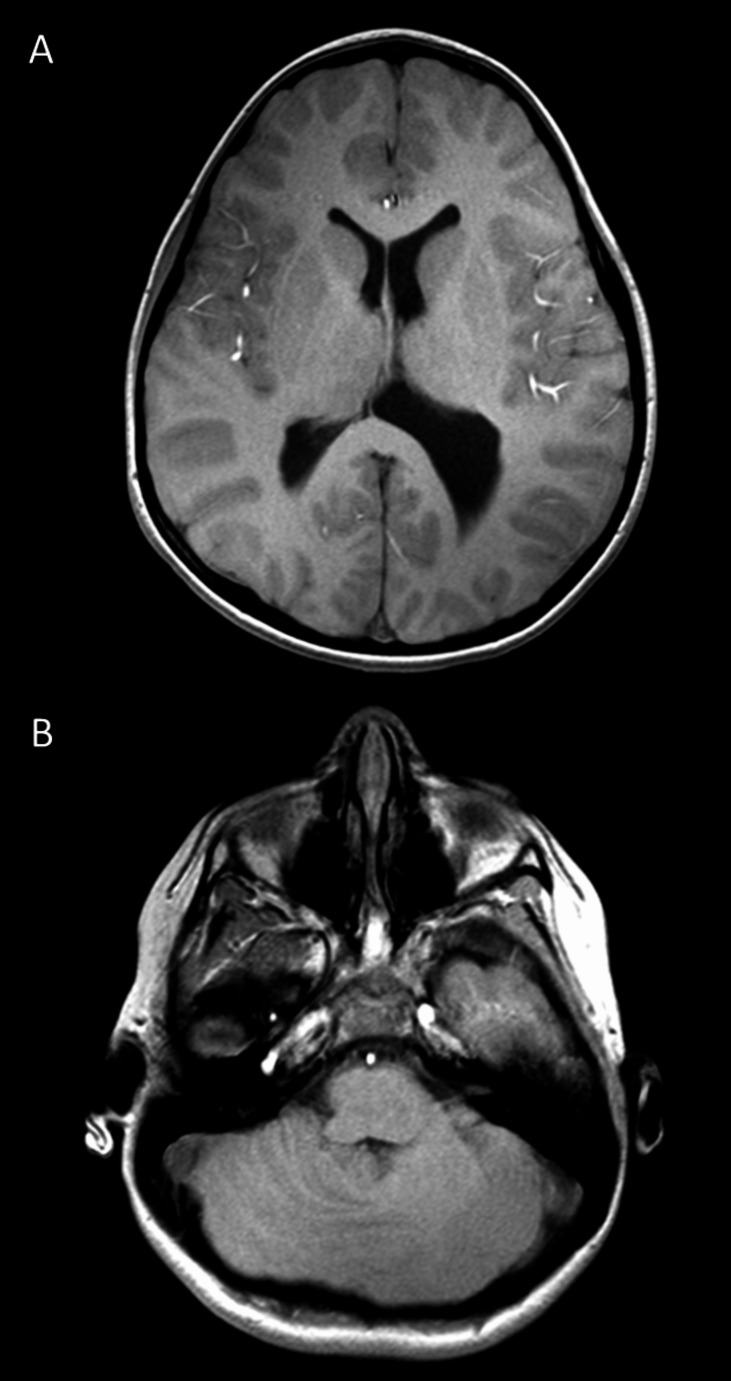
T1-weighted axial 3T MRI scan at the age of 8 years showing left ventriculomegaly (**A**) and fusion of the cerebellar hemispheres (**B**)

At 5 months, she developed a sensorimotor anomaly of the binocular vision system in which the foveal line of alignment of one eye occasionally deviated inward and failed to intersect the object of fixation (i.e., alternating esotropia). After patching and wearing glasses, strabismus surgery was performed at 16 months. Her development was characterized by hypotonia with delayed attainment of motor milestones for which she received occupational and physical therapy. She also received speech therapy for low tone in her mouth and jaw. She drooled all the time. Postural instability, perceptual difficulties, and poor gross and fine motor skills were noted. At approximately 7 years of age, she had an episode of repeated tongue chewing to the point that she developed an ulcer. In addition, she developed an oral allergy syndrome and her tongue began to itch extremely and relentlessly. She was put on divalproex sodium, which effectively eliminated the tongue chewing. Magnetic resonance imaging of the brain at 8 years of age showed left ventriculomegaly, especially of the left temporal and occipital horns (Fig. 1A) and identified RES (Fig. 1B).

Concomitant ^99m^Tc-HMPAO single photon emission computed tomography (SPECT) showed moderately elevated relative perfusion of the bilateral orbitofrontal cortex. A marked elevation of relative perfusion was seen in the right insular cortex, as well as in the anterior and posterior cingulate cortex, adjacent precuneus area, right caudate head, and to a lesser extent the bilateral putamen. A focal area of marked elevated relative perfusion was also observed in the superior aspect of the left cerebellar hemisphere. By contrast, marked relative underperfusion was found in the left mesial temporal lobe.

Neuropsychological evaluation performed at age 7 showed that the patient fell in the high-average psychometric range of intelligence with a WISC-4 full scale IQ of 112. There were initially no indications of verbal or reading deficits, and also no signs of deficits in factual learning and declarative memory. However, the presence of inattentiveness, distractibility, impulsive behaviour, and emotion dysregulation fulfilled the Diagnostic and Statistical Manual of Mental Disorders fourth edition (DSM-IV) characteristics of attention-deficit (hyperactivity) disorder (ADHD). Notably, she did not meet diagnostic criteria for autism. At 12 years of age, she underwent a renewed evaluation of psychological and social-emotional functioning. Results of the examination revealed that according to the DSM-IV criteria along with the results of the prior assessment, she met diagnostic criteria for ADHD, depressive disorder, generalized anxiety disorder, and developmental coordination disorder. In addition to experiencing a considerable number of problematic thoughts, ideas, and feelings, her emotional reactions were strong and at the same time variable and unpredictable. Difficulties in emotion regulation were considered the underlying cause of the intensity, unpredictability, changeability, and inconsistency of her behaviour. Moreover, she exhibited an external locus of control as she believed that events beyond her control drove her actions and outcomes. This constellation of signs and symptoms is reminiscent of CCAS [8, 11].

Between 9 and 12 years of age several stimulants were trialed to manage her ADHD symptoms. Methylphenidate (10–20 mg) was started at about 9 years of age and continued for more than 2 years. Despite the positive effect, medication was terminated due to poor weight gain. A dextroamphetamine-amphetamine stimulant caused headache and nausea and was discontinued after 2 days. A methylphenidate transdermal patch did not improve symptoms and was stopped. Another trial of methylphenidate and atomoxetine was without success. Guanfacine, an adrenergic alpha-2 receptor agonist, did not seem to have a positive effect but was nonetheless continued given the positive clinical evidence in children and adolescents with ADHD (e.g., [12]). At 12 years of age the selective serotonin reuptake inhibitor (SSRI) escitalopram (10 mg) was started, which was stopped due to hyperactivity and restlessness. A trial of the SSRI citalopram (30 mg) when she was 13 years old was also deemed unsuccessful.

Due to reading problems and persistent postural instability, oculomotor and vestibular testing were performed. Results revealed abnormal conjugate eye movements and abnormalities in visuo-vestibular interactions, both of which are consistent with involvement of the oculomotor vermis and its target in the posterior portion of the fastigial nucleus [13]. As she progressed into college, an evaluation of her strengths and weaknesses was performed at the age of 18. At that time, she was taking the following drugs on a daily basis: Guanfacine (2 mg) and methylphenidate (15 mg in the morning and 5 mg at midday as needed) for ADHD, divalproex sodium (1000 mg) for tongue chewing, a serotonin-norepinephrine reuptake inhibitor (100 mg) for anxiety, vitamin D to help with anxiety and depressive mood, melatonin (5 mg) for sleeping difficulties, anti-histamines (10 mg) for allergies, probiotics and the food supplements zinc, magnesium, vitamin A, and co-enzyme Q10 (50 mg).

Results of the Behavior Rating Inventory of Executive Functioning that was administered when she was 18 years old showed significant problems in her ability to inhibit impulses, shift to new situations, and maintain emotional control. She frequently experienced intense emotions, which she found difficult to regulate. From the cognitive viewpoint, she had the tendency to get ‘stuck’ in the moment, showing difficulties in implementing subsequent steps toward resolving a conflict or negotiating challenge. Difficulties in executive functioning were seen in solving time-efficiency problems and rapid processing of visual information. By contrast, she demonstrated extraordinary language and strong conversation skills. She was emotionally insightful, articulate, and well-liked by adults and her peers. She made friends easily, but found it hard to maintain relationships as she experienced abnormally heightened levels of anxiety in regard to social interactions. Moreover, she was and still is highly talented in the arts, including acting, dancing, and singing.

**Fig. 2:**
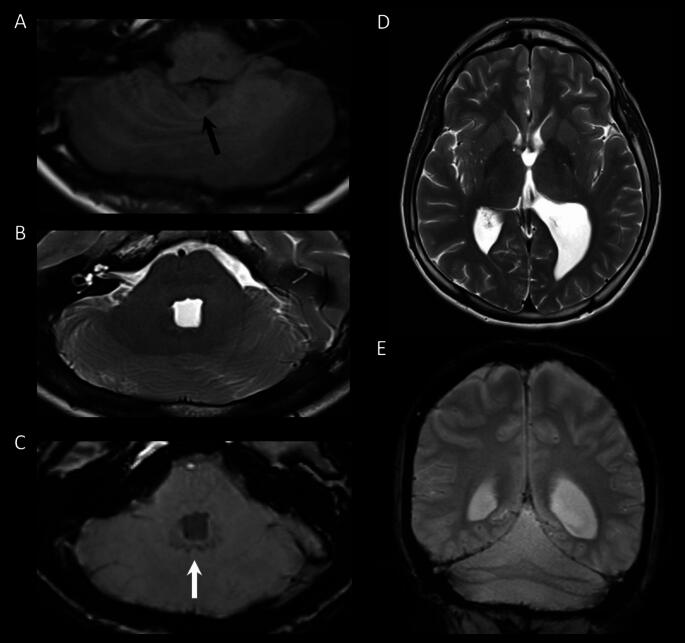
Axial T1-weighted MRI scan at the age of 22 years showing an absent vermis with the nodulus (black arrow) still present (**A**). T2-weighted axial image showing the fused cerebellar hemispheres, an abnormally square-shaped fourth ventricle, and mild flattening of the ventral pons (**B**). Axial susceptibility-weighted image depicting an area of hypointense signal crossing the midline dorsal to the fourth ventricle (white arrow) indicating dentate nuclei fusion (**C**). T2-weighted axial image visualizing the asymmetric enlargement of the left lateral ventricle with dilation especially of the left temporal and occipital horn (**D**). Coronal multi-echo gradient-recalled echo T2*-weighted image showing dilation of the left occipital horn and fused cerebellar hemispheres (**E**)

Currently, at the age of 22, a repeated, contrast enhanced 3T MRI scan demonstrates a stable RES condition, normal grey-white matter distinction, and no signs of malformations of the cerebral hemispheres. There is no indication of interhemispheric cerebral fusion, no midline fusion of the colliculi in the midbrain, and no abnormalities in the basal ganglia and thalamus. The septum pellucidum is present. In the infratentorial brain, mild flattening of the ventral pons can be observed. The cerebellum shows nearly complete fusion of the cerebellar hemispheres across the midline (Fig. 2A and E). The anterior and posterior vermis are absent, but a distinct nodulus is present (Fig. 2A). The hypointense signal across the midline present in the susceptibility-weighted image indicates that the dentate nuclei are fused (Fig. 2C). The shape of the fourth ventricle is enlarged and square-shaped on the axial view (Fig. 2B). As a result of the ventriculomegaly, a mild upward bowing of the corpus callosum was noted, but without signs of thinning or dysgenesis.

CCAS-related neuropsychiatric symptoms have persisted up to this day and include emotional reactivity, emotion dysregulation, anxiety, attentional problems, impulsivity, and impairments in executive functioning. In fact, with increasing age her tics and emotions appear to have gotten harder to manage. Also, her obsessive thoughts and compulsive behaviours, as well as anxiety, have become worse. To manage her symptoms related to the tics and anxiety she takes the benzodiazepine alprazolam on a regular basis. Furthermore, her mother noticed a growing lack of social awareness as she does not always seem to read social cues well and understand what appropriate conversation is.

Notwithstanding her strengths, continued support remains necessary to maintain her cognitive and socio-emotional functioning in daily life activities. For example, stress management (e.g., deep breathing exercises and mindfulness), cognitive behavioural therapy methods, acceptance and/ or commitment therapy to address anxiety management and for developing effective coping skills focussed on adaptive problem solving. These forms of support may help to decrease her emotional reactivity, improve symptom management of tic behaviours, and acquire skills to help recognize changes in mood or an increase in anxiety.

## Discussion

The role of the cerebellar vermis in emotional reactivity, emotion dysregulation, impulsive behaviour, and anxiety is well-documented in both neurotypical individuals and cerebellar patients [14]. The constellation of neuropsychiatric abnormalities in our patient with a developmental (posterior) vermian anomaly adds to this growing body of literature and provides further evidence that the (midline) cerebellum is an important hub in brain networks subserving non-motor functions. In addition, anxiety has also been linked to the vestibulocerebellar system, wherein abnormal eye movements and the experience of nausea and vertigo are part of a vermal-fastigial dysregulation syndrome [15].^1^

The hypothesized role of the cerebellum in emotion regulation and executive function is further supported by a previous report of a male adolescent with RES and similar clinical issues who had no apparent extracerebellar structural abnormalities [7, 16]. Both individuals fit the CCAS profile associated with congenital and acquired vermis abnormalities that can lead to a variety of neuropsychiatric symptoms. In addition to the features mentioned above, CCAS can include flattening of affect, mood disturbances, pathological laughter and crying, impulsivity, and aggression [17]. Furthermore, the tongue chewing as observed in our patient could potentially reflect a rare form of self-mutilation as described in another RES patient who was diagnosed with an obsessive-compulsive personality disorder associated with emotional instability and oral self-mutilation [18].

The intense emotional reactions that are simultaneously variable and unpredictable concur with the universal cerebellar transform (UCT) view. The UCT conceptualizes the cerebellum as a multimodal oscillation dampener which rapidly and automatically optimizes performance according to context [19]. That is, analogous to impairments in the rate, rhythm, and accuracy of movements, cerebellar damage can cause disruptions in the timing, intensity, and appropriateness of emotions and actions (i.e., dysmetria of thought) [20]. Moreover, the UCT and dysmetria of thought hypothesis may also provide a framework for understanding her anxiety, tics, and ADHD symptoms in terms of managing context-appropriate thoughts and behaviour.

The cerebellum may best be viewed as a structure that is part of functional brain networks dedicated to cognition and affect [21]. Connections between the medial cerebellum and the reticular system, amygdala, periaqueductal grey, ventral tegmental area, and hypothalamus provide examples of neuroanatomical routes for the vermis to partake in the experience and regulation of emotions and motivated behaviour [17]. Impairments in attention, executive functioning, and processing speed, flat affect, and moderate-to-severe clinical depression were recently reported in a 44-year-old female with a solitary tumour in the culmen of the cerebellar vermis. A SPECT scan revealed hypoperfusion in the left frontal lobe [22]. After tumour resections, substantial improvement in cognitive and affective symptoms was seen within a week together with complete regression of the oedema and normalization of left frontal lobe perfusion [22]. Furthermore, results of another SPECT study in medication-naïve children with ADHD showed bilateral cerebellar hypoperfusion in the posterolateral hemispheres (Crus II) [23]. The cerebellar hypoperfusion was paralleled by decreased perfusion in the medial orbitofrontal gyrus, middle frontal gyrus, and temporal gyrus, as well as increased perfusion in the posterior somatosensory cortices [23]. A more recent study in treatment-naïve adult ADHD patients found that connectivity disruptions within the cerebellum correlated with hyperactive-impulsive and inattentive behaviour [24]. White matter aberrations in the cerebellum of RES patients may point towards abnormal signal transfer between the cerebellum and the rest of the brain [25, 26]. However, contributions of the cerebellum to understand the neuropsychiatric symptoms of our patient remain speculative. For example, the left ventriculomegaly may explain the hypoperfusion in the mesial temporal lobe and emotional symptoms associated with amygdala dysfunction. Temporal lobe abnormalities have also been linked to impulsivity, hyperorality, and problems in emotion recognition, as well as prosodic comprehension (cf. Klüver-Bucy syndrome). Additionally, divalproex sodium has the potential to exert negative psychological side-effects, including irritability, aggression, hypersensitivity, and attention deficits [27]. It is therefore possible that the long-time use of divalproex sodium contributes to the neuropsychiatric symptoms of our patient. Aside from possible side effects of medication, neuropsychiatric symptoms could also be potentially linked to familial inheritance.

Finally, compensatory mechanisms including cerebellar reorganisation and recruitment of extracerebellar regions, as perhaps in part illustrated by the earlier SPECT findings, can be readily expected. However, cognitive, emotional, and social difficulties have remained fairly constant during the patient’s development from childhood to young adulthood. Actually, neuropsychiatric symptoms, including tics, obsessive compulsive behaviour, and anxiety, appear to have worsened in recent years. The increase in symptoms may be indicative of heightened levels of psychological stress and cognitive challenges associated with young adulthood, such as the fact that she is now living on her own. The alleged involvement of the cerebellum in emotion regulation and the view that the cerebellum is intrinsically part of the brain’s stress circuit. This circuit includes reciprocal monosynaptic connections between the vermis and hypothalamus in the mammalian brain [28], fitting the idea that cerebellar abnormalities can predispose to disruptions in emotion reactivity and coping style [29]. Longitudinal and standardized assessment of CCAS will be informative to monitor changes in neuropsychiatric symptomatology of RES and other cerebellar patients over time [10, 30].

To conclude, while neuropsychiatric symptoms tended to vary somewhat over the years, emotional reactivity and dysregulation, impulsivity, anxiety, and impairments in executive functioning can be considered as the most consistent and relevant clinical features of our patient. The observed neuropsychiatric symptoms fit those seen in congenital and acquired focal cerebellar abnormalities and have been described as a collection of abnormal cerebellar interactions with cerebral cortical (e.g., prefrontal, parietal, and temporal cortices), striatal, and limbic (e.g., amygdala, hypothalamus, and anterior cingulate cortex) areas of the brain [15]. The presence of cerebellar pathology is proposed to constitute a vulnerability factor for experiencing cognitive, social, and emotional problems in daily life. Neuropsychiatric symptoms associated with RES may intensify during psychological stress and patients seem to be at higher risk of developing mental health issues. Further systematic research is needed to elucidate the proposal of dysfunctional cerebello-extracerebellar networks in RES.

## Data Availability

The data that support the findings of this report are available upon reasonable request.

## Footnotes

1 In clinical ataxiology CCAS is considered a separate syndrome next to the cerebellar motor syndrome and the vestibulo-cerebellar syndrome [9]. However, a degree of inter-relatedness can nonetheless be expected.

## References

[1] Sukhudyan B, Jaladyan V, Melikyan G, Schlump JU, Boltshauser E, Poretti A. Gómez-López-Hernández syndrome: reappraisal of the diagnostic criteria. Eur J Pediatr. 2010;169:1523–8. 10.1007/s00431-010-1259-7. 20652311 10.1007/s00431-010-1259-7

[2] Fernández-Jaén A, Fernández-Mayoralas DM, Calleja-Pérez B, Muñoz-Jareño N, Moreno N. Gomez-Lopez-Hernandez syndrome: two new cases and review of the literature. Pediatr Neurol. 2009;40(1):58–62. 10.1016/j.pediatrneurol.2008.10.001. 19068257 10.1016/j.pediatrneurol.2008.10.001

[3] Ng R, Richard AE, Heinrich K. Developmental risk for mood dysregulation in a pediatric case of Gómez-López-Hernández syndrome: neurocognitive considerations. Clin Neurol Neurosurg. 2020;195:105877. 10.1016/j.clineuro.2020.105877. 32454266 10.1016/j.clineuro.2020.105877

[4] Gomez MR. Cerebellotrigeminal and focal dermal dysplasia: a newly recognized neurocutaneous syndrome. Brain Dev. 1979;1:253–6. 10.1016/S0387-7604(79)80039-X. 95427 10.1016/s0387-7604(79)80039-x

[5] Ishak GE, Dempsey JC, Shaw DW, Tully H, Adam MP, Sanchez-Lara PA, Glass I, Rue TC, Millen KJ, Dobyns WB, Doherty D. Rhombencephalosynapsis: a hindbrain malformation associated with incomplete separation of midbrain and forebrain, hydrocephalus and a broad spectrum of severity. Brain. 2012;135(Pt 5):1370–86. 10.1093/brain/aws065. 22451504 10.1093/brain/aws065 PMC3338925

[6] Fouda MA, Kim TY, Cohen AR, Rhomboencephalosynapsis. Review of the literature. World Neurosurg. 2022;159:48–53. 10.1016/j.wneu.2021.12.062. 34954057 10.1016/j.wneu.2021.12.062

[7] Tully HM, Dempsey JC, Ishak GE, Adam MP, Mink JW, Dobyns WB, Gospe SM Jr, Weiss A, Phillips JO, Doherty D. Persistent figure-eight and side-to-side head shaking is a marker for rhombencephalosynapsis. Mov Disord. 2013;28(14):2019–23. 10.1002/mds.25634. 24105968 10.1002/mds.25634 PMC5510988

[8] Schmahmann JD, Sherman JC. The cerebellar cognitive affective syndrome. Brain. 1998;121(Pt 4):561–79. 10.1093/brain/121.4.561. 9577385 10.1093/brain/121.4.561

[9] Manto M, Mariën P. Schmahmann’s syndrome - identification of the third cornerstone of clinical ataxiology. Cerebellum Ataxias. 2015;2:2. 10.1186/s40673-015-0023-1. 26331045 10.1186/s40673-015-0023-1 PMC4552302

[10] Hoche F, Guell X, Vangel MG, Sherman JC, Schmahmann JD. The cerebellar cognitive affective/Schmahmann syndrome scale. Brain. 2018;141(1):248–70. 10.1093/brain/awx317. 29206893 10.1093/brain/awx317 PMC5837248

[11] Koziol LF, Budding D, Andreasen N, D’Arrigo S, Bulgheroni S, Imamizu H, Ito M, Manto M, Marvel C, Parker K, Pezzulo G, Ramnani N, Riva D, Schmahmann J, Vandervert L, Yamazaki T. Consensus paper: the cerebellum’s role in movement and cognition. Cerebellum. 2014;13(1):151–77. 10.1007/s12311-013-0511-x. 23996631 10.1007/s12311-013-0511-x PMC4089997

[12] Biederman J, Melmed RD, Patel A, McBurnett K, Konow J, Lyne A, Scherer N, SPD503 Study Group. A randomized, double-blind, placebo-controlled study of guanfacine extended release in children and adolescents with attention- deficit/hyperactivity disorder. Pediatrics. 2008;121(1):e73–84. https://doi.org10.1542/peds.2006-3695. 10.1542/peds.2006-3695 18166547

[13] Kheradmand A, Zee DS. Cerebellum and ocular motor control. Front Neurol. 2011;2:53. 10.3389/fneur.2011.00053. 21909334 10.3389/fneur.2011.00053 PMC3164106

[14] Adamaszek M, D’Agata F, Ferrucci R, Habas C, Keulen S, Kirkby KC, Leggio M, Mariën P, Molinari M, Moulton E, Orsi L, Van Overwalle F, Papadelis C, Priori A, Sacchetti B, Schutter DJ, Styliadis C, Verhoeven J. Consensus Paper: cerebellum and emotion. Cerebellum. 2017;16(2):552–76. 10.1007/s12311-016-0815-8. 27485952 10.1007/s12311-016-0815-8

[15] Schmahmann JD, Weilburg JB, Sherman JC. The neuropsychiatry of the cerebellum - insights from the clinic. Cerebellum. 2007;6(3):254–67. 10.1080/14734220701490995. 17786822 10.1080/14734220701490995

[16] Schutter DJLG, Paalman M, Henssen D, Deschamps PKH. A case of attention deficit hyperactivity disorder in rhombencephalosynapsis. Cerebellum. 2021;20(4):659–61. 10.1007/s12311-021-01234-x. 33590442 10.1007/s12311-021-01234-x PMC8360900

[17] Schmahmann JD. The role of the cerebellum in affect and psychosis. J Neurolinguistics. 2000;13(2–3):189–214. 10.1016/S0911-. 6044(00)00011 – 7.

[18] Verri A, Uggetti C, Vallero E, Ceroni M, Federico A. Oral self-mutilation in a patient with rhombencephalosynapsis. J Intellect Disabil Res. 2000;44(Pt 1):86–90. 10.1046/j.1365-2788.2000.00242.x. 10711654 10.1046/j.1365-2788.2000.00242.x

[19] Schmahmann JD. From movement to thought anatomic substrates of the cerebellar contribution to cognitive processing. Hum Brain Mapp. 1996;4(3):174–98. 10.1002/(SICI)1097-0193(1996)4. 20408197 10.1002/(SICI)1097-0193(1996)4:3<174::AID-HBM3>3.0.CO;2-0

[20] Schmahmann JD. An emerging concept. The cerebellar contribution to higher function. Arch Neurol. 1991;48(11):1178–87. 1953406 10.1001/archneur.1991.00530230086029

[21] Talmasov D, Schmahmann JD. Cognition, emotion, and cerebrocerebellar circuit disruption in a patient with hemangioblastoma. Neurology. 2023;101(12):507–8. 10.1212/WNL.0000000000207626. 37407267 10.1212/WNL.0000000000207626

[22] Inoue M, Oya S, Yamaga T, Tajima T, Hanakita S. Pearls & oysters: cognitive and affective dysfunction caused by a small cerebellar hemangioblastoma. Neurology. 2023;101(12):e1272–5. 10.1212/WNL.0000000000207509. 37407260 10.1212/WNL.0000000000207509 PMC10516281

[23] Lee JS, Kim BN, Kang E, Lee DS, Kim YK, Chung JK, et al. Regional cerebral blood flow in children with attention deficit hyperactivity disorder: comparison before and after methylphenidate treatment. Hum Brain Mapp. 2005;24:157–64. 10.1002/hbm.20067. 15486990 10.1002/hbm.20067 PMC6871721

[24] Ohnishi T, Toda W, Itagaki S, Sato A, Matsumoto J, Ito H, Ishii S, Miura I, Yabe H. Disrupted structural connectivity and less efficient network system in patients with the treatment-naive adult attention-deficit/hyperactivity disorder. Front Psychiatry. 2023;14:1093522. 10.3389/fpsyt.2023.1093522. 37009101 10.3389/fpsyt.2023.1093522 PMC10061975

[25] Merlini L, Fluss J, Korff C, Hanquinet S. Partial rhombencephalosynapsis and Chiari type II malformation in a child: a true association supported by DTI tractography. Cerebellum. 2012;11(1):227–32. 10.1007/s12311-011-0300-3. 21833660 10.1007/s12311-011-0300-3

[26] Widjaja E, Blaser S, Raybaud C. Diffusion tensor imaging of midline posterior fossa malformations. Pediatr Radiol. 2006;36(6):510–7. 10.1007/s00247-006-0146-x. 16708205 10.1007/s00247-006-0146-x

[27] National Health Care Institute. (2024). Valproic acid side effects. Retrieved from https://www.farmacotherapeutischkompas.nl/bladeren/preparaatteksten/v/valproinezuur#bijwerkingen. Accessed April 26, 2024.

[28] Haines DE, Dietrichs E, Mihailoff GA, McDonald EF. The cerebellar-hypothalamic axis: basic circuits and clinical observations. Int Rev Neurobiol. 1997;41:83–107. 10.1016/s0074-7742(08)60348-7. 9378614 10.1016/s0074-7742(08)60348-7

[29] Schutter DJLG. The cerebellum in emotions and psychopathology. London: Routledge Knowledge; 2020.

[30] Maas RPPWM, Killaars S, van de Warrenburg BPC, Schutter DJLG. The cerebellar cognitive affective syndrome scale reveals early neuropsychological deficits in SCA3 patients. J Neurol. 2021;268(9):3456–66. 10.1007/s00415-021-10516-7. 33743045 10.1007/s00415-021-10516-7 PMC8357713

